# Reconstructing the Gait Pattern of a Korean Cadaver with Bilateral Lower Limb Asymmetry Using a Virtual Humanoid Modeling Program

**DOI:** 10.3390/diagnostics15151943

**Published:** 2025-08-02

**Authors:** Min Woo Seo, Changmin Lee, Hyun Jin Park

**Affiliations:** 1 Department of History, College of Liberal Arts, Sejong University, Seoul 05000, Republic of Korea; seominwoo40@gmail.com; 2 Future City and Society Research Institute, Yonsei University, Seoul 03722, Republic of Korea; lcmin@yonsei.ac.kr; 3 Department of Anatomy, School of Medicine, Konkuk University, Chungju 27478, Republic of Korea

**Keywords:** gait analysis, limb length discrepancy (LLD), femoral fracture, Meta Motivo

## Abstract

**Background and Objective**: This study presents a combined osteometric and biomechanical analysis of a Korean female cadaver exhibiting bilateral lower limb bone asymmetry with abnormal curvature and callus formation on the left femoral midshaft. **Methods**: To investigate bilateral bone length differences, osteometric measurements were conducted at standardized landmarks. Additionally, we developed three gait models using *Meta Motivo*, an open-source reinforcement learning platform, to analyze how skeletal asymmetry influences stride dynamics and directional control. **Results**: Detailed measurements revealed that the left lower limb bones were consistently shorter and narrower than their right counterparts. The calculated lower limb lengths showed a bilateral discrepancy ranging from 39 mm to 42 mm—specifically a 6 mm difference in the femur, 33 mm in the tibia, and 36 mm in the fibula. In the gait pattern analysis, the normal model exhibited a straight-line gait without lateral deviation. In contrast, the unbalanced, non-learned model demonstrated compensatory overuse and increased stride length of the left lower limb and a tendency to veer leftward. The unbalanced, learned model showed partial gait normalization, characterized by reduced limb dominance and improved right stride, although directional control remained compromised. **Conclusions**: This integrative approach highlights the biomechanical consequences of lower limb bone discrepancy and demonstrates the utility of virtual agent-based modeling in elucidating compensatory gait adaptations.

## 1. Introduction

Gait analysis is of considerable significance in both clinical and rehabilitative fields, as human locomotion represents a highly complex integration of multiple physiological, biomechanical, and behavioral factors [1,2,3]. Gait analysis method can be widely divided into two categories: motion/appearance-based and model-based [4,5]. Motion/appearance-based analysis, which uses silhouettes as fundamental data, is conducted by using various templates (Gait Energy Image, Gait Flow Image, Active Energy Image, Frame Difference Energy Image) and sequences (GaitSet, Gaitpart) for gait recognition [1,5]. However, these methods not only fail to adequately account for the periodicity of gait but are also highly susceptible to external factors such as ground contact and clothing, making it difficult to analyze the intrinsic characteristics of gait itself [5].

In contrast, model-based analysis offers the advantage of robustness to changes in external factors, as it collects gait information by modeling skeletal data, body structure, or motion patterns [5]. Given the multifactorial complexity of human gait, which encompasses biomechanical, neuromuscular, and musculoskeletal components, OpenSim and the AnyBody Modeling System have been widely adopted as two prominent advanced computational platforms for gait analysis, enabling the generation and evaluation of dynamic movement simulations to elucidate neuromuscular coordination, gait cycle dynamics, internal mechanical loading, athletic performance metrics, and the biomechanical implications of human motion [6,7]. Especially, there has been a growing number of studies in recent years utilizing deep learning for model-based gait analysis [8,9].

This study investigates the cause and estimated timing of unilateral lower limb bone shortening observed during anatomical dissection of a medical cadaver, which was associated with a pathological lesion in the left femur. By analyzing osteometric data from both lower limb bones, the individual’s antemortem gait was reconstructed to explore the relationship between limb length discrepancy (LLD) and abnormal gait patterns. Notably, this study employed *Meta Motivo*, an open-source reinforcement learning platform for training humanoid agents, in the gait reconstruction process. This represents the first application of this program in gait reconstruction research, highlighting its potential utility and applicability in future studies. Furthermore, this study is expected to contribute to the understanding of pathological gait patterns in patients with skeletal pathologies and aid in their early diagnosis.

## 2. Materials and Methods

### 2.1. Cadaveric Specimen

**Figure 1:**
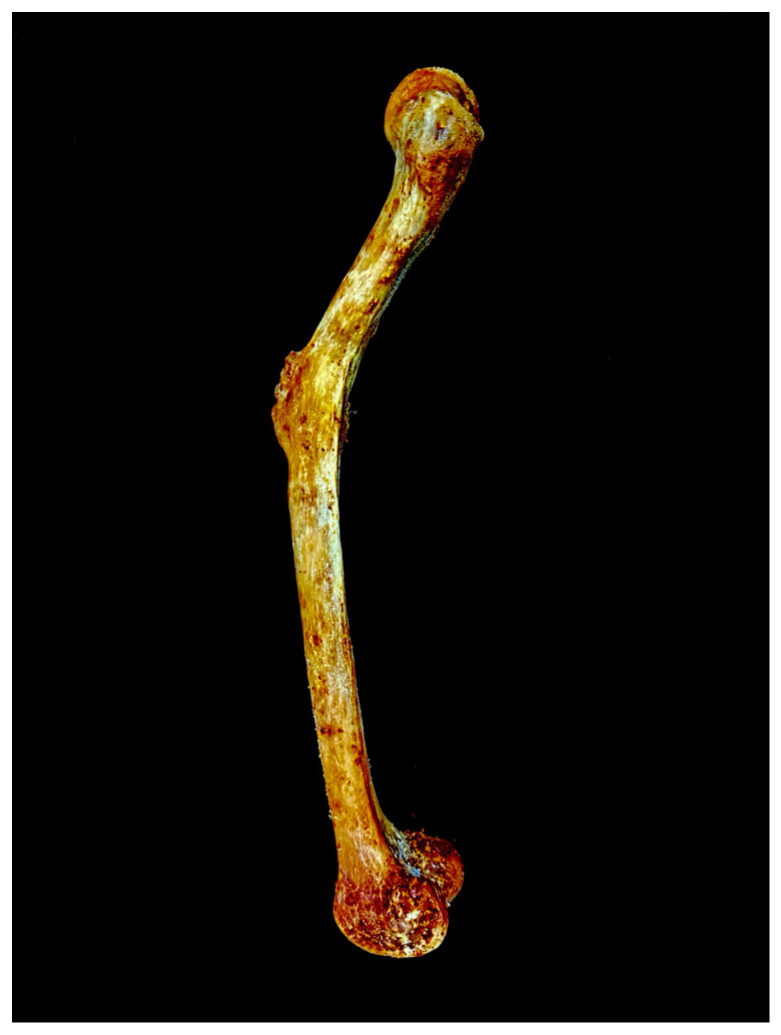
Left femur in the sagittal plane. Abnormal curvature and callus formation are observed in the midshaft region.

During routine dissection of a Korean female cadaver (age 59, 151 cm), abnormal curvature and callus formation were observed in the midshaft of the left femur (Figure 1). This study was conducted in accordance with the Declaration of Helsinki ethical principles for medical research involving human subjects. The body was legally donated to Sangji University College of Korean Medicine.

**Figure 2:**
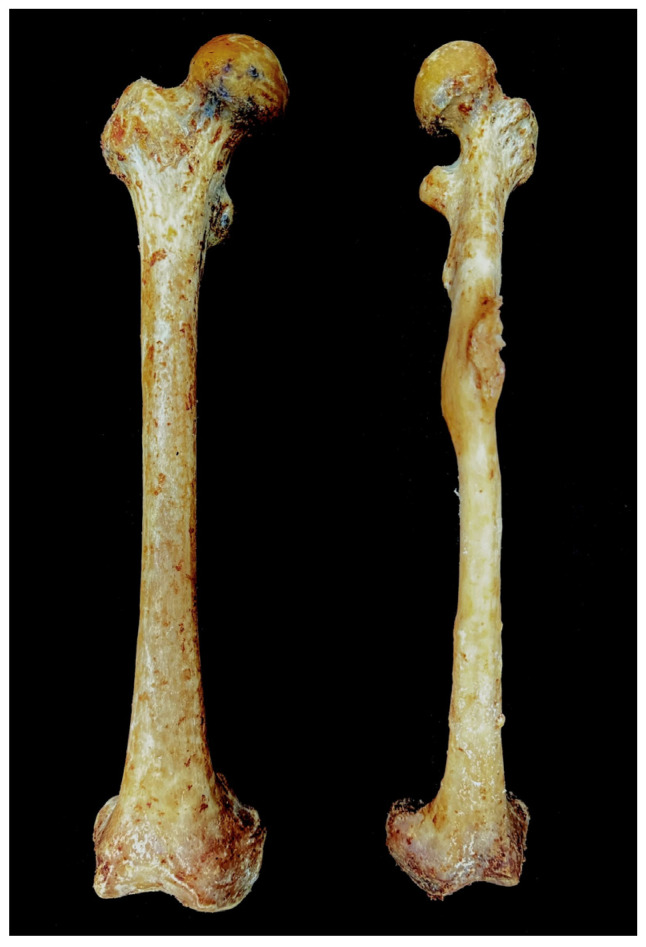
Bilateral femur in the anterior view. The right femur shows no macroscopic signs of trauma or fracture and retains normal length compared to its left counterpart.

A healed fracture of the left femur, presumed to have resulted from inadequate treatment during life, exhibits curvature and callus formation in the midshaft region. The remainder of the left femoral midshaft surface appears smooth and well-remodeled, indicating substantial post-fracture bone healing. In comparison, the right femur displays no macroscopic signs of trauma or fracture and retains normal length (Figure 2). Notably, the left femur is significantly shorter than the right, further supporting the impact of the previous fracture and subsequent healing process. The pronounced severe curvature of the femur and the sequential shortening of the lower limb bones resulting from the femoral lesion illustrate the pathological uniqueness and rarity of this case.

For an accurate comparison between the normal and abnormal femurs, and to assess any potential effects on other lower limb bones, the maceration process was conducted following the method of Park et al. (2024) [10]. After disarticulation at each joint and maximal removal of soft tissues, lower limb bones were completely immersed and simmered for 12 h in a thermostatic water bath (WBT-45, Jeongbio, Incheon, Republic of Korea) containing 30 L of 10% oxygen-based bleach (Yuhangen^®^, Yuhan, Seoul, Republic of Korea) at 194 °F (90 °C). After simmering, residual soft tissues were carefully removed using a periosteal elevator. To eliminate residual moisture from within the bones, the specimen was immersed in ethanol for 24 h and then air-dried under ambient conditions.

### 2.2. Osteometrical Analysis

To find the metrical differences, five parameters for the femur, four parameters for the tibia, three parameters for the patella, and two parameters for the fibula (Table 1) were recorded using a digital sliding caliper (Mitutoyo, Tokyo, Japan) and an osteometric board (Ward’s Science, New York, NY, USA).

### 2.3. Meta Motivo Analysis

For the gait analysis, *Meta Motivo*—an open-source platform designed to simulate and train virtual humanoid agents with diverse form factors [11]—was used. We present three gait models developed with *Meta Motivo* to evaluate its capacity for generating faithful, temporally coherent locomotion under varying osteological conditions. First, a baseline humanoid—possessing osteologically normal, bilaterally symmetrical lower limb bones—was trained to reproduce normative walking patterns. Second, we instantiated an osteologically validated asymmetric variant by incorporating a shortened left leg, thereby introducing skeletal imbalance. Third, this asymmetric model was retrained using *Meta Motivo*’s dual reward scheme—combining *motion-tracking* and *forward-backward consistency* objectives—to maximize both kinematic fidelity and temporal coherence.

**Table 1:** Oseometrical parameters for each lower limb bone [12,13,14].

Bones	Parameters	Reference
Femur	Maximum length	Martin and Knussmann, 1988 [12]
	Head diameter	Moore-Jansen et al., 1994 [13]
	Midshaft transverse diameter	Moore-Jansen et al., 1994 [13]
	Midshaft sagittal diameter	Moore-Jansen et al., 1994 [13]
	Epicondylar breadth	Martin and Knussmann, 1988 [12]
Patella	Maximum length	Byrd and Adams, 2003 [14]
	Breadth	Byrd and Adams, 2003 [14]
	Thickness	Byrd and Adams, 2003 [14]
Tibia	Maximum length	Martin and Knussmann, 1988 [12]
	Proximal epiphyseal breadth	Martin and Knussmann, 1988 [12]
	Distal epiphyseal breadth	Martin and Knussmann, 1988 [12]
	Nutrient foramen transverse diameter	Moore-Jansen et al., 1994 [13]
Fibula	Maximum length	Martin and Knussmann, 1988 [12]
	Midshaft diameter	Martin and Knussmann, 1988 [12]

The *motion tracking reward* constituted a pivotal component that quantifies how accurately the agent reproduces behavioral trajectories derived from observed data (e.g., motion-capture data). During training, the agent’s instantaneous state (e.g., joint angles, angular velocities, spatial positions, etc.) is continuously compared against the target trajectory, and smaller deviations yield higher rewards.

The *forward-backward consistency* reward acts as a regularization term that enforces logical coherence of the agent’s policy in both forward and backward time directions. Specifically, it penalizes discrepancies between the policy used to advance along the target trajectory in the forward direction and the policy reconstructed when traversing the trajectory in reverse. By minimizing this bidirectional policy divergence, the reward structure ensures that the agent does not merely imitate instantaneous behaviors at individual time steps but instead learns a unified, connected sequence of actions. Consequently, the resulting control policy exhibits enhanced stability and generalizability across extended time horizons. This mechanism enables the agent to produce more natural, continuous behaviors even in unobserved states or under external perturbations during training, thereby establishing a foundation for zero-shot performance across diverse downstream tasks [11].

## 3. Results

### 3.1. Osteometric Results

**Figure 3:**
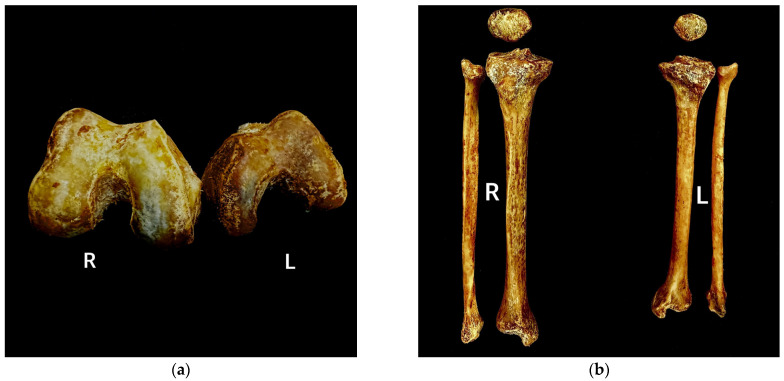
Distal epiphyses of bilateral femur (**a**) and bilateral patella, tibia, and fibula (**b**). All parts of the pathology-affected left lower limb bones are consistently shorter than those of the right side.

The skeletal measurements marked notable asymmetry between the right and left lower limb bones, with the left side exhibiting consistently smaller results across all parameters (Table 2). The femur demonstrated an average bilateral difference of 6.8 mm across the four measured parameters, with the most pronounced asymmetry observed in epicondylar breadth, which exhibited a 14 mm disparity between the left and right sides (Figure 3a).

Although the patella constitutes the smallest element of the lower limb bones, it exhibited a mean bilateral difference of 8 mm across the three measured parameters, surpassing the average asymmetry observed in the femur. The tibia and fibula also demonstrated markedly greater bilateral asymmetry than the femur, with mean side-to-side differences of 14 mm and 19.5 mm, respectively (Figure 3b). Notably, both the tibia and fibula exhibited pronounced bilateral discrepancies in maximum length, with inter-limb differences of 39 mm and 42 mm, respectively.

The estimated lower limb length, derived from the sum of the maximum lengths of the lower limb long bones, revealed a substantial bilateral discrepancy ranging from 39 mm to 42 mm, suggesting that the specimen may have experienced impaired locomotion during life.

Especially for the bilateral difference in breadth of the knee joint parts: femoral epicondyle (14 mm), patella (13 mm), and proximal tibial epiphysis (12 mm) suggest a coordinated asymmetry, reflecting structural biomechanical interdependence within the patellofemoral-tibial complex.

### 3.2. Gait Analysis

The gait analysis scenarios were systematically compared across three conditions: Normal (symmetric lower limb lengths), unbalanced, non-learned (asymmetric lower limb lengths without adaptive learning), and unbalanced, learned (asymmetric lower limb lengths with adaptive learning).

#### 3.2.1. Normal

The case of symmetrical and normal lower limb lengths has been shown to result in a straight-line gait, with no observable lateral deviation during walking.

#### 3.2.2. Unbalanced: Non-Learned

**Figure 4:**
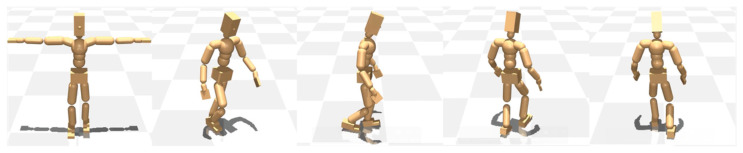
Gait patterns for the unbalanced, non-learned case. The model shows a marked reliance on the left leg, a dragging motion of the right leg, and the overall deviation of the walking trajectory and the gait posture to the left (a dynamic illustration is available in the Supplementary Materials as Video S1).

The model demonstrates a predominant reliance on the left lower limb during ambulation. This compensatory mechanism results in an increased stride length on the left. Conversely, the right lower limb exhibits a shortened stride length and a characteristic dragging motion during the swing phase of the gait cycle. The model’s overall posture during gait is noticeably tilted toward the left side (Figure 4).

Regarding gait direction, the model exhibits impaired directional control during ambulation, characterized by an inability to maintain a linear trajectory and a tendency to veer into a broader circular pattern toward the left.

#### 3.2.3. Unbalanced: Learned

**Figure 5:**
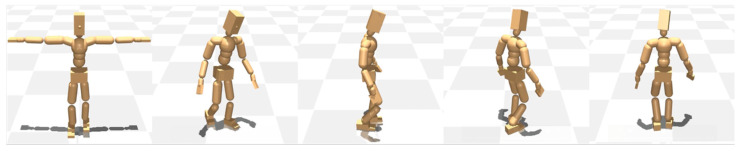
Gait patterns for the unbalanced, learned case. The model shows a noticeable reduction in excessive reliance on the left leg and the degree of right leg dragging, indicating a partial shift toward a more normalized gait compared to the Unbalanced: Non-learned case (a dynamic illustration is available in the Supplementary Materials as Video S2).

While the overall gait pattern remains similar to that observed in the second condition, a noticeable reduction in the model’s excessive reliance on the left leg and the degree of right leg dragging was observed, indicating a partial shift toward a more normalized gait. This was accompanied by a shift in stride dynamics, marked by a reduction in the left side stride length and a compensatory increase in the right stride length. However, the model’s gait posture and directional pattern remain largely consistent with those observed in the second condition (Figure 5).

## 4. Discussion

### 4.1. Cause of the Femoral Curvature and Its Timing

**Figure 6:**
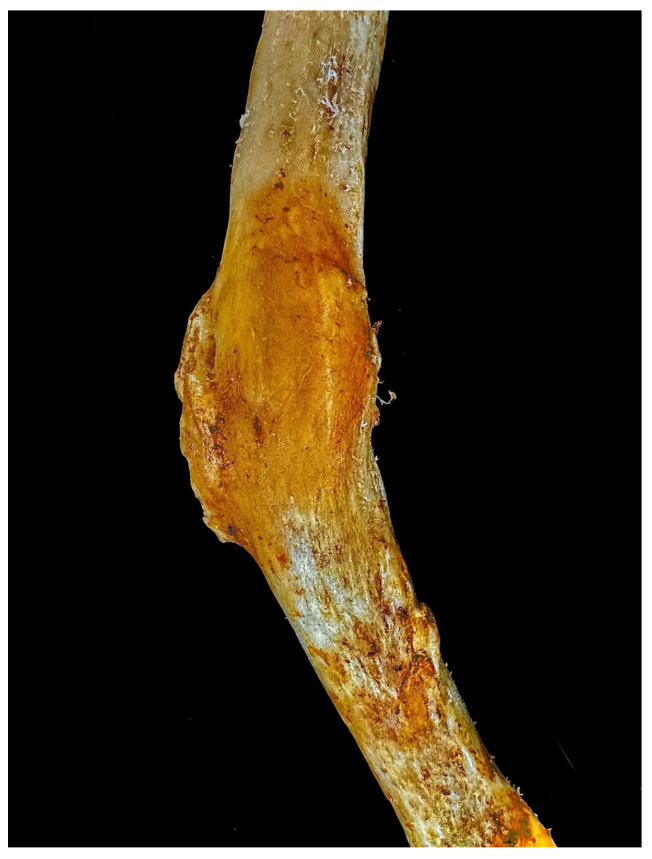
Midshaft of the left femur with fracture traces. The midshaft region shows well-healed fracture traces and callus formation, suggesting an antemortem origin and considerable antiquity.

In this study, we examined the morphological and morphometric changes in the femur exhibiting abnormal curvature, as well as those of other lower limb bones that may be associated with femoral pathology. The abnormal midshaft curvature of the femur, which is highly likely to have been caused by femoral fracture, could potentially have originated from various pathologies, including biochemical stress [15], metabolic bone disorders such as Paget’s disease [16] and osteomalacia [17], and chronic pathologies such as poliomyelitis [18]. Although the exact cause and timing of the curvature can be diagnosed through bone tissue analysis using imaging techniques such as CT scans, the presence of well-healed fracture traces (Figure 6) and the consistently shorter and smaller morphology of the left patella, tibia, and fibula compared to their right counterparts strongly suggest that the fracture occurred long before death and that the individual experienced significant functional impairment due to the injury during life. Although the healing process of femoral midshaft fracture typically takes about 3 or 4 weeks in infancy and 12 to 16 weeks during adolescence [19], the observed sequential shortening of the lower limb bones indicates that the trauma likely occurred during the individual’s developmental years, suggesting antemortem origin and considerable antiquity.

The orthopedic literature has reported that femoral shortening due to malunion or growth plate injuries in pediatric fractures triggers sequential underdevelopment of other lower limb bones [20,21]. This pathomechanical pattern parallels observations in neuromuscular disorders such as post-polio syndrome, where chronic disuse and muscular atrophy induce proportional thinning and shortening of the femur, tibia, and fibula [18,22]. Given this established mechanism, it is plausible to infer that the consistent deformities observed in the left lower limb bones in this case may have resulted from a pediatric fracture.

Furthermore, the abnormal curvature observed at the midshaft of the left femur suggests that a fracture sustained during childhood underwent inadequate clinical management. Notably, callus formation occurs during the fracture healing process, and the dimension or type of callus can differ depending on the timing and resolution of the different stages of fracture healing [19,23]. A complete and well-formed callus observed in this case indicates that the bone fragments have undergone sufficient remodeling to achieve clinical stability and strength. Therefore, if this remodeling progressed in the context of malalignment, it is highly probable that the femur underwent plastic deformation, resulting in permanent angular distortion as the bone consolidated in a misaligned orientation.

### 4.2. Gait Patterns

Limb length discrepancy (LLD), while potentially attributable to various functional deformities involving abnormal movements of the hip, knee, ankle, or foot, mostly originates from true bony leg differences, an osteological difference between the lengths of the two limbs from the femoral head to the distal tibia [24,25]. Although LLD is common in both healthy and pathological conditions [26], it causes gait disorders in both contexts. Previous literature has mentioned that gait deviations occur when the discrepancy is over 20 mm [25,26], so we can consider that a bilateral difference of 39 mm causes serious gait disturbance.

The gait disturbance in this case can be corroborated through analysis using *Meta Motivo*, which reveals a marked reliance on the left leg, a dragging motion of the right leg, and an overall deviation of the walking trajectory and gait posture to the left. Considering that previous literature has revealed that greater functional emphasis is placed on the shorter limb in individuals as a compensatory strategy [27], the results of the present study appear to represent the typical gait patterns observed in patients with LLD, alsdn0. Moreover, deviated posture during the swing phase is likely the result of multiple compensatory strategies, including increased hip and knee joint flexion in the longer limb and increased ankle joint plantar flexion knee and hip joint extension in the shorter limb [25].

LLD is likely to result in differences not only in gait patterns but also in other physiological activities. The study by Gurney et al. (2001) [28], which investigated the effects of LLD on gait economy and lower extremity muscle activity in older adults, found that oxygen consumption and the rating of perceived exertion were greater in 2 cm LLD. Notably, a significant increase in heart rate, minute ventilation, and quadriceps activity was observed in 3 and 4 cm LLD, and plantar flexor activity in 4 cm LLD. Considering that the LLD in this case measures 3.9 cm, the likelihood of the individual developing the aforementioned physiological disorders is considerably elevated. Moreover, an individual is highly likely to be exposed to metabolic disorders such as obesity, diabetes, and hyperlipidemia induced by decreased physical activity and chronic pain in the musculoskeletal system, lower extremities, and lower back caused by postural instability [29].

### 4.3. Limitations

In this study, due to the inability to perform CT scans or similar imaging techniques, the skeletal structure exhibiting pathological phenomena could not be investigated, and thus the precise causes or pathological manifestations could not be identified.

In the gait analysis, gait disturbance patterns were analyzed solely based on osteometric perspective. However, considering that gait is a complex phenomenon influenced by multiple factors such as muscle strength, joint range of motion, and the body’s adaptive mechanisms, this approach inevitably represents a simplified interpretation that does not fully capture gait patterns. Furthermore, since it was not possible to verify the individual’s gait during life, an accurate comparison of the analyzed gait patterns was challenging. We aim to conduct further research that comprehensively incorporates the biomechanical mechanisms of the neuromuscular system and utilizes advanced gait analysis programs to achieve a more precise evaluation of individual gait patterns.

## Figures and Tables

**Table 2:** Measurements for each lower limb bone parameter.

Bones	Parameters	Results (mm)	
		Right	Left
Femur	Maximum length	400	394
	Head diameter	47	43
	Midshaft transverse diameter	27	24
	Midshaft sagittal diameter	24	17
	Epicondylar breadth	83	69
Patella	Maximum length	41	33
	Breadth	54	41
	Thickness	19	16
Tibia	Maximum length	331	298
	Proximal epiphyseal breadth	75	63
	Distal epiphyseal breadth	48	45
	Nutrient foramen transverse diameter	29	21
Fibula	Maximum length	332	296
	Midshaft diameter	16	13

## Data Availability

The data presented in this study are available on request from the corresponding author.

## Supplementary Materials

The following supporting information can be downloaded at https://www.mdpi.com/article/10.3390/diagnostics15151943/s1, Video S1: Gait patterns for unbalanced, non-learned case; Video S2: Gait patterns for unbalanced, learned case.
